# Biotransformation of Polyphenols in Apple Pomace Fermented by β-Glucosidase-Producing *Lactobacillus rhamnosus* L08

**DOI:** 10.3390/foods10061343

**Published:** 2021-06-10

**Authors:** Lihua Liu, Chenyi Zhang, Huimin Zhang, Guoqiang Qu, Chun Li, Libo Liu

**Affiliations:** 1 Institute of Animal Sciences, Chinese Academy of Agricultural Sciences, Beijing 100093, China; liulihua01@caas.cn; 2 Key Laboratory of Dairy Science, College of Food Science, Northeast Agricultural University, Harbin 150030, China; klds@neau.edu.cn (C.Z.); spxylch@gmail.com (H.Z.); dupeng@neau.edu.cn (G.Q.); lchun@neau.edu.cn (C.L.); 3 Heilongjiang Green Food Research Institute, Harbin 150028, China

**Keywords:** apple pomace, by-product, phenolic compounds, lactic acid bacteria, β-glucosidase

## Abstract

Apple pomace, the main by-product in apple processing, is a cheap source of bioactive compounds that could be used in the food industry. However, the value of this by-product is still far from being fully realized. In this study, 11 strains of *Lactobacillus* strains were assayed for β-glucosidase activity, and only *Lactobacillus* *rhamnosus* L08 (*L. rhamnosus* L08) showed high cell-membrane associated β-glucosidase activity. We then evaluated the effects of fermentation of apple pomace using the selected strain, focusing on the biotransformation of polyphenols and antioxidant capacity. We found that *L. rhamnosus* L08 fermentation significantly reduced the contents of quercitrin and phlorizin in apple pomace, while increasing the contents of quercetin and phloretin. The contents of gallic acid, epicatechin acid, caffeic acid, and ferulic acid were also increased in apple pomace after fermentation. In addition, the antioxidant activities of apple pomace were enhanced during fermentation, based on the bioconversion of phenolic profiles. Our results demonstrate that lactic acid bacteria fermentation is a promising approach to enhance the bioactivity of phenolic compounds in apple pomace. Moreover, this study demonstrates that, as a valuable processing by-product with bioactive components, apple pomace can be used in the food industry to provide economic benefits.

## 1. Introduction

Fruits are processed into juice, wine, canned fruits, and other products in the food industry, generating a large amount of agricultural waste during these processes, including peel, seeds, and small amounts of flesh [1]. Apples, one of the most consumed fruit crops in the world, produces a by-product known as apple pomace that accounts for 25%–30% of the whole fruit weight in the production of apple juice and cider [2]. Apple pomace is usually used as animal feed, but a larger amount of apple pomace is still discarded and wasted, resulting in adverse impacts on the environment and sustainable agricultural development [3]. Thus, the efficient recovery and utilization of apple pomace could contribute to reducing food waste and generating economic and environmental benefits due to its low cost and readily availability.

Studies have shown the potential applications of apple pomace as stabilizers and nutrient fortifiers in food products. Wang, Kristo, and LaPointe [4] reported that apple pomace powder can improve the stability of yogurt during storage periods. The addition of apple pomace obviously increased the dietary fiber content and antioxidant properties in meat products [5]. Despite this, the value of apple pomace is still far from being fully realized in the food industry, particularly considering the presence of natural bioactive compounds that can be used in functional foods [6].

Apple pomace is an abundant source of bioactive compounds, owing to high amounts of functional compounds in apples, which are present in its peels [3,7], particularly phenolics, which are well-known for their various beneficial effects on human health, such as antioxidant, antimicrobial, anti-inflammatory, and anti-tumor properties [8,9]. The polyphenols of apple pomace are mainly flavonoids (such as quercetin and phloretin), followed by phenolic acids (such as caffeic acid, chlorogenic acid, and catechin) [10]. Bound polyphenols account for more than half of the total phenol content, affecting their antioxidant properties, whereas their aglycones, produced by hydrolysis, tend to exhibit higher antioxidant capacity [11,12].

Many studies have shown that lactic acid bacteria (LAB) fermentation can extend the shelf-life of fruits and enrich their nutritional value (such as that of apples, pomegranates, and mulberries), especially the bioavailability and bioactivity of phytochemicals [13,14], and can also increase the content of potentially functional microbial metabolites [15]. The conversion of glycosides into their aglycone form in plant-based foods during fermentation by LAB with high β-glucosidase activity is responsible for their antioxidant capacity [16]. Wu et al. [17] reported that apple juice is an excellent substrate for LAB growth, and LAB fermentation improved the production of ketones and aldehydes and enriched the aroma complexity in apple juice. A recent study by Li et al. [18] showed that LAB fermentation altered phenolic profiles in jujube juices prepared from two crop varieties, thereby improving its antioxidant capacities. However, the literature on the fermentation of apple pomace with LAB featuring high β-glucosidase activity is still limited.

In this study, we investigated the effects of *Lactobacillus rhamnosus* L08 (*L. rhamnosus* L08) fermentation on apple pomace, focusing on the biotransformation of polyphenols and antioxidant capacity. In addition, the properties of β-glycosidase production in the selected *L. rhamnosus* L08 were studied. Our data demonstrate that LAB fermentation is a promising approach to enhancing the bioactivity of phenolic compounds in apple pomace. Furthermore, our results encourage the use of apple pomace as a potential source of phenolic compounds, thereby providing economic benefits for the apple processing industry.

## 2. Materials and Methods

### 2.1. Chemicals

De Man, Rogosa, Sharpe (MRS) broth and Esculin-MRS agar medium was purchased from Sharing Technologies (Shanghai, China). Potassium ferricianyde, ferric chloride, ferrous sulfate, trichloroacetic acid, salicylic acid, and methanol (High Performance Liquid Chromatography, HPLC grade) were purchased from Tianli Biochemical Co., Ltd. (Tianjin, China). 2,2-diphenyl-1-picrylhydrazyl (DPPH) and Folin–Ciocalteus reagent were purchased from Biotopped Life Sciences (Beijing, China). Gallic acid, epicatechin acid, ferulic acid, caffeic acid, quercitrin, quercetin, phlorizin, phloretin, and p-nitrophenyl-β-D-glucopyranoside were purchased from Sigma-Aldrich (Beijing, China). All other reagents were purchased from Basifu Co., Ltd. (Tianjin, China).

### 2.2. Bacterial Strains and Culture Conditions

Four strains of *Lactobacillus plantarum* (903, 904, 905, 960), four strains of *Lactobacillus acidophilus* (810, 812, 813, 866), and three strains of *Lactobacillus rhamnosus* (L06, L07, L08) were obtained from the Key Laboratory of Dairy Science (Harbin, China). Bacterial cultures were stored in MRS medium supplementing with glycerol (20%, *v*/*v*) at −80 °C. For activation, the strains were inoculated (2%, *v*/*v*) into 10 mL of MRS broth and then incubated at 37 °C for 16 h in a temperature-controlled incubator. Then, the screening of β-glucosidase-producing strains was carried out as described by Choi, Kim, Kim, and Shin [19] using Esculin-MRS agar medium.

### 2.3. β-Glucosidase Activity

The β-glucosidase-producing strain was selected for further studies in this work. Firstly, the β-glucosidase producing strain was cultured in MRS medium at 37 °C for 12 h and harvested by centrifugation (6000× *g*, 10 min, 4 °C), and washed twice with phosphate buffer saline (PBS, 0.1 M, pH 7.0). Then, the whole-cell concentrate, cell-free extracts, and cell-membrane extracts were prepared for analysis of β-glucosidase activity according to the method of Ávila et al. [20] with some modifications. Briefly, 0.2 mL of p-nitrophenyl-β-D-glucopyranoside (1 mM), 0.8 mL of PBS (0.1 M, pH 5.0), and 0.2 mL of an enzyme solution were added to the test tubes and incubated at 40 °C for 30 min. The reaction was stopped by the addition of 1 mL of sodium carbonate solution (1 M). The amount of p-nitrophenol formed was measured spectrophotometrically at 405 nm using a microplate reader (SpectraMax i3x, Molecular Devices, San Jose, CA, USA). One enzyme unit was defined as the amount of enzyme that released 1 μmoL p-nitrophenol from the substrate per min.

### 2.4. Effects of Culture Conditions on β-Glucosidase Production

The effects of culture conditions, including culture time, temperature, and pH, on the β-glucosidase activity of the selected strain were evaluated as follows: 20 mL of bacterial cells were inoculated into a 250-mL Erlenmeyer flask containing 100 mL of MRS medium and cultured at 37 °C, then the β-glucosidase activity was measured every 2 h for 24 h. The effect of temperature on β-glucosidase activity was evaluated by maintaining the temperature of the culture medium at 30 °C, 33 °C, 37 °C, 40 °C, and 43 °C. For analyzing the effect of culture pH on β-glucosidase activity, the medium pH values were adjusted to 5.0, 5.5, 6.0, 6.5, and 7.0.

### 2.5. Fermentation of Apple Pomace

Fuji apples were obtained from a local fruit supermarket (Harbin, China) to collect apple pomace. The apple pomace (consisting of peel and core) was washed with potable water and blended with water in proportions of 1:2 (*w*/*w*) using a sanitized food processor. Then, apple pomace was pasteurized at 72 °C for 15 min and pH was adjusted to 6.0 using sodium carbonate for the subsequent fermentations. After cooling to room temperature, the selected strain cells (2%, *v*/*v*) were inoculated into 250 mL of apple pomace (in 500-mL Erlenmeyer flasks) and then incubated at 37 °C for 7 d in a temperature-controlled incubator.

### 2.6. Total Phenolic Content (TPC)

TPC was determined using the Folin–Ciocalteu method with some modifications [17]. Briefly, 0.5 mL of standard or the diluted sample (1:10) was mixed with 2.5 mL of Folin–Ciocalteu reagent (10%, *w*/*v*) for 5 min in darkness, and 2 mL of sodium carbonate (7.5%, *w*/*v*) was subsequently added to the mixture. The absorbance was measured at 760 nm using a microplate reader after the above mixture was incubated in the dark at 40 °C for 1 h. Results were present as gallic acid equivalent (μg/mL).

### 2.7. Quantification of Polyphenols in Fermentation Apple Pomace by HPLC

**Figure 1:**
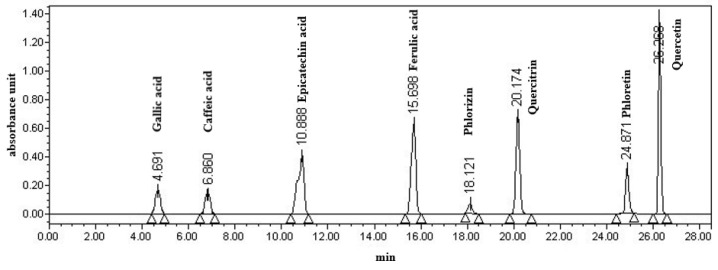
Chromatogram of 8 polyphenols detected with HPLC at 280 nm.

Apple polyphenols were determined as described by Wu et al. [17] with minor modifications. Eight phenolic standards (gallic acid, epicatechin acid, ferulic acid, caffeic acid, quercitrin, quercetin, phlorizin, and phloretin) were used for calibration curves. Twenty-five milliliters of apple fermentation broth were added with the same amount of ethyl acetate for extraction, and then the extracted solutions were merged for rotary evaporation at 35 °C. The extracted polyphenols were dissolved in 10 mL of chromatography-grade anhydrous ethanol for HPLC analysis. The separation was performed on an Agilent C18 (5 μm, 4.6 × 250 mm) column (Agilent Technology Co., Ltd., Beijing, China). The mobile phase A was composed of water with 0.01% of phosphoric acid and the mobile phase B was methanol. The gradient program was as follows: 0–20 min, 20%–50% solvent B; 20–25 min, 50%–70% solvent B; 25–30 min, 70%–80% solvent B; 30–35 min, 20% solvent B. The HPLC detection parameters were as follows: a UV wavelength of 280 nm, a flow rate of 1 mL/min, a column temperature of 30 °C, an injection volume of 20 μL (Figure 1).

### 2.8. Determination of Antioxidant Activities

#### 2.8.1. DPPH Radical Scavenging Activity

The DPPH radical scavenging activity was measured using the method of Kwaw et al. [14] with some modifications. Briefly, 20 mL of apple pomace fermentation broth were centrifuged at 6000× *g* for 10 min at 4 °C to collect the supernatant. Then, 1 mL of the supernatant was mixed with 2 mL of DPPH solution (0.2 mM) prepared in methanol. The mixture was incubated at room temperature for 30 min in darkness and then the absorbance was measured at 517 nm using a microplate reader. Results were calculated using the following equation:

(1) $$\mathrm{DPPH}\;\mathrm{radical}\;\mathrm{scavenging}\;\mathrm{activity}\;\left(\%\right)=\left(1-\frac{A_{s}-A_{b}}{A_{c}}\right)$$

where *A_s_*, *A_b_*, and *A_c_* were the absorbance of the sample with DPPH solution, the sample with methanol, and the control (without sample), respectively.

#### 2.8.2. Hydroxyl Radical Scavenging Activity

Hydroxyl radical scavenging activity was measured according to the method of He et al. [21] with minor modifications. Twenty milliliters of apple pomace fermentation broth were centrifuged at 6000× *g* for 10 min at 4 °C to collect the supernatant. Then, 1 mL of the supernatant was mixed with 1 mL of ferrous sulphate (9 mM), 1 mL of salicylic acid (9 mM) prepared in ethanol, and 1 mL of hydrogen peroxide (8.8 mM). The mixture was incubated at 37 °C for 30 min and then the absorbance was measured at 510 nm using a microplate reader. Results were calculated using the following equation:

(2) $$\mathrm{Hydroxyl}\;\mathrm{radical}\;\mathrm{scavenging}\;\mathrm{activity}\;\left(\%\right)=\left(1-\frac{A_{s}-A_{b}}{A_{c}}\right)$$

where *A_s_* is the absorbance of the sample, *A_b_* is the absorbance of the sample without hydrogen peroxide, and *A_c_* is the absorbance of the control (without sample).

#### 2.8.3. Reducing Power Assay

The reducing power assay was performed according to a method previously reported by Natic et al. [22] with some modifications. Twenty milliliters of apple pomace fermentation broth were centrifuged at 6000× *g* for 10 min at 4 °C to collect the supernatant. Then, l mL of the supernatant was mixed with 0.05 mL of HCl (0.01 M), 0.4 mL of potassium ferricyanide (0.02 M), 0.4 mL of FeCl_3_ (0.02 M), and 0.7 mL of distilled water. The absorbance was measured at 700 nm after the mixture was incubated at 37 °C for 30 min in the dark.

### 2.9. Statistical Analysis

Data are presented as the means with standard deviations (SD) from three independent experiments. Statistical analysis was carried out with a one-way ANOVA using Statistical Product and Service Solutions 22.0 (SPSS, Inc., Chicago, IL, USA) and GraphPad Prism 8 (GraphPad Software, San Diego, CA, USA). Correlation analysis was performed by using the Pearson correlation coefficient. A *p*-value of < 0.05 was considered to be statistically significant.

## 3. Results and Discussion

### 3.1. Screening for Strains Producing β-Glucosidase

**Figure 2:**
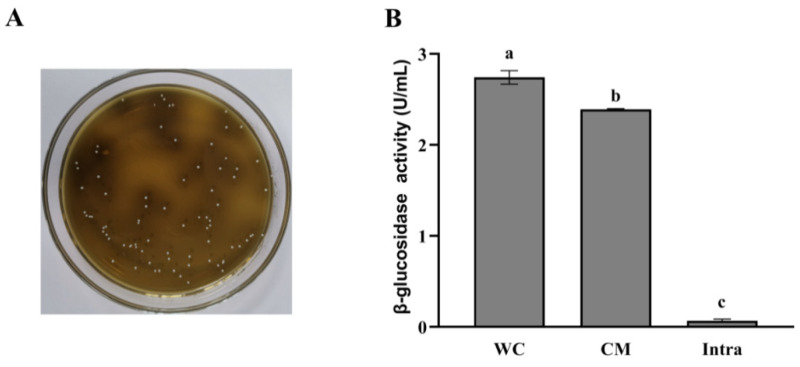
(**A**) Production of β-glucosidase from *Lactobacillus rhamnosus* L08 detected in Esculin-MRS agar medium; (**B**) subcellular location of β-glucosidase in *Lactobacillus rhamnosus* L08. WC: whole-cell concentrates, CM: cell-membrane extracts, intra: intracellular extracts. Data are means ± SD of three biological replicates. Bars labeled with different letters are significantly different (*p* < 0.05).

We screened the strains producing β-glucosidase using Esculin-MRS agar medium, according to the principle that strains producing β-glucosidase can hydrolyze esculin to its aglycone, which reacts with Fe^3+^, appearing as colonies surrounded by a dark brown zone [19]. As shown in Table 1 and Figure 2A, among all strains tested, only *L. rhamnosus* L08 showed black colonies, demonstrating that this strain can produce β-glucosidase. Therefore, *L. rhamnosus* L08 was chosen for apple pamoce fermentation. Then, we determined the subcellular location of glycosidase activities in *L. rhamnosus* L08 (Figure 2B). The β-glucosidase activities of the whole-cell concentrate, cell-membrane extracts, and intracellular extracts were 2.74, 2.39, and 0.08 U/mL, respectively. The obtained results clearly showed that β-glucosidase seemed to be cell membrane-bound in *L. rhamnosus* L08.

β-glucosidase is an important enzyme that can be used in the conversion of flavonoid glycosides into their aglycone form with more biological activity [23]. In general, the difference in β-glucosidase production may be attributed to strain specificity and growth conditions [24]. Ávila et al. [20] evaluated that the β-glucosidase activity of 15 *Lactobacillus* strains, most of which are associated with the cell-envelope and the enzyme activity, ranged from 0.08 to 2.18 U/mL, which is in agreement with our result.

**Figure 3:**
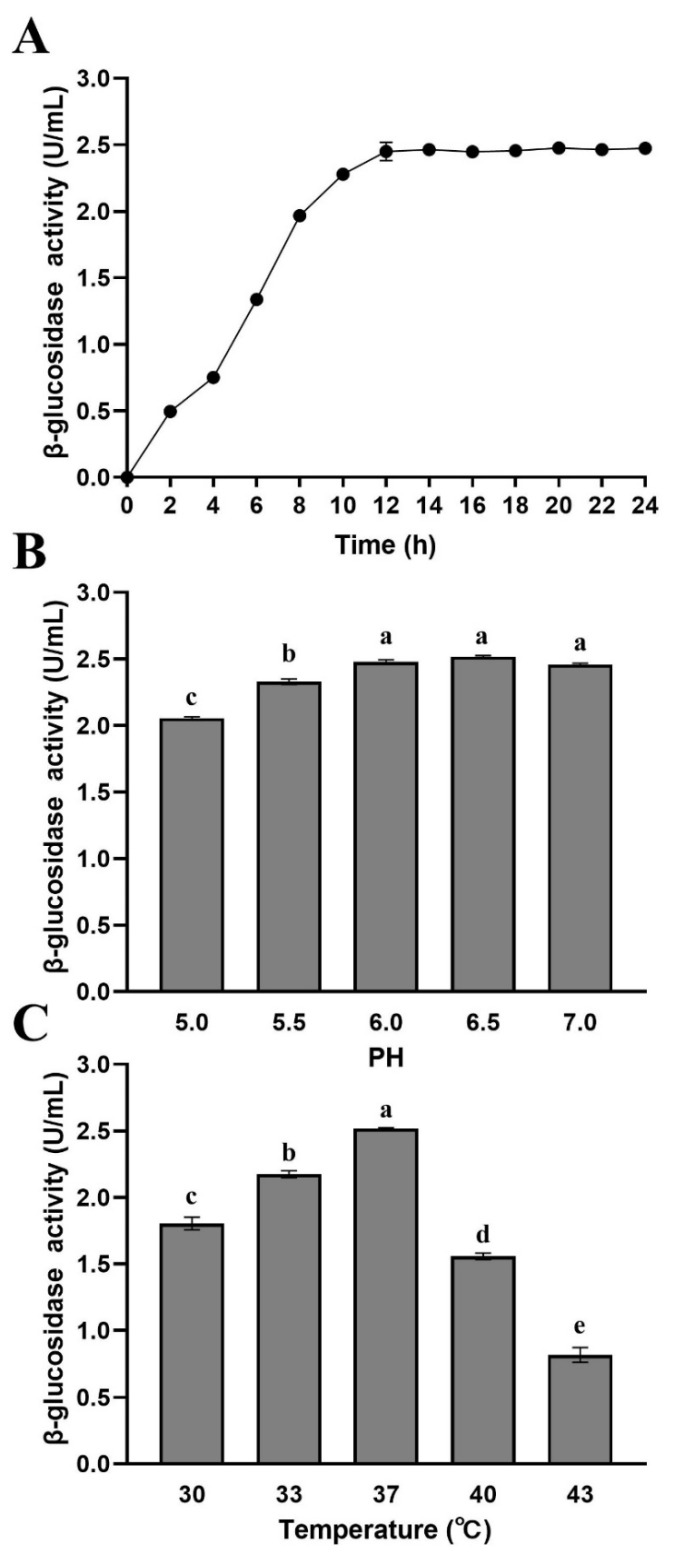
Effects of culture conditions on the β-glucosidase activity of *Lactobacillus rhamnosus* L08. (**A**) Time, (**B**) pH, and (**C**) temperature. Data are means ± SD of three biological replicates. Different letters above bars indicate significant differences (*p* < 0.05).

Figure 3 shows the effects of growth time, pH, and temperature on the β-glucosidase production of *L. rhamnosus* L08. The β-glucosidase activity of *L. rhamnosus* L08 reached a maximum value of 2.47 U/mL (Figure 3A). The β-glucosidase activity first increased at 0–12 h and then remained stable over time, apparently corresponding to the strain growth, because the β-glucosidase in *L. rhamnosus* L08 was associated with the bacterial cell membrane. The β-glucosidase activity of *L. rhamnosus* L08 ranged from 2.06 to 2.52 U/mL under different culture pH levels, and the highest enzyme activity was noted at pH 6.5 (Figure 3B). In addition to the cultivation pH, the growth temperature is also important factor affecting the growth and the glycosidase production of bacteria [25]. We observed a significant difference in enzyme activity between different culture temperatures (*p* < 0.05) (Figure 3C). When the culture temperature was higher than 37 °C, the enzyme activity decreased rapidly. The highest β-glucosidase activity (2.61 U/mL) was obtained at 37 °C. These results indicated that the optimal conditions, namely, temperature (37 °C) and pH (6.5), for the growth of *L. rhamnosus* L08 were favorable for β-glucosidase production.

### 3.2. TPC during Fermentation

The phytochemical concentrations in fruits or vegetables during the LAB fermentation process are generally affected by the fermentation substrate, strains, and fermentation time [26]. Wu et al. [17] reported that total phenols in apple juice fermented by several *Lactobacillus* strains decreased to varying degrees during 48 h of fermentation. Vivek, Mishra, Pradhan, and Jayabalan [27] observed that total polyphenols and anthocyanins were increased in fruit juice during 72 h of fermentation with *Lactobacillus plantarum*.

**Figure 4:**
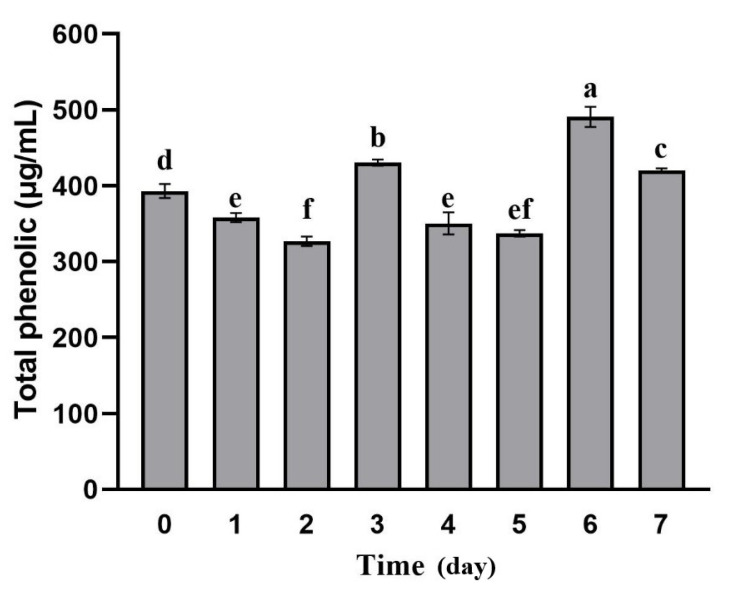
Changes in total phenol content of apple pomace during fermentation. Data are means ± SD of three biological replicates. Bars with different letters differ significantly (*p* < 0.05).

In this study, we analyzed changes of TPC in apple pomace during fermentation (Figure 4). We observed a significant increase in TPC at 3 (440.45 μg/mL) and 6 days (480.80 μg/mL) compared to unfermented apple pomace (383.00 μg/mL) (*p* < 0.05). The TPC of apple pomace at 3 and 6 days was increased by 15.00% and 25.54%, respectively. The change of TPC can be explained by bacterial growth and glycosidase activity during fermentation [28]. The hydrolysis of complex polyphenols into simpler forms conducted by the strain adapted to the fermentation environment, resulting in an increase of the TPC at 3 days of fermentation. After 4 to 5 days of fermentation, the decrease in the polyphenol content maybe due to the strain utilizing some phenolic compounds as a carbon source to maintain their own growth. The significant increase in polyphenol content at 6 days may be due to the accumulation of β-glucosidase, produced during the fermentation of *Lactobacillus rhamnose* L08, which further catalyzes the hydrolysis of complex polyphenols, resulting in more active phenolic compounds [28]. According to Capanoglu et al. [29], the TPC determination using the Folin–Ciocalteu assay was affected by some reductants, such as ascorbic acid, citric acid, simple sugars, or certain amino acids. Although *L. rhamnosus* L08 fermentation can increase the TPC in pomace, the actual result may not be as high as that observed in this study.

### 3.3. Phenolic Profiles during Fermentation

**Figure 5:**
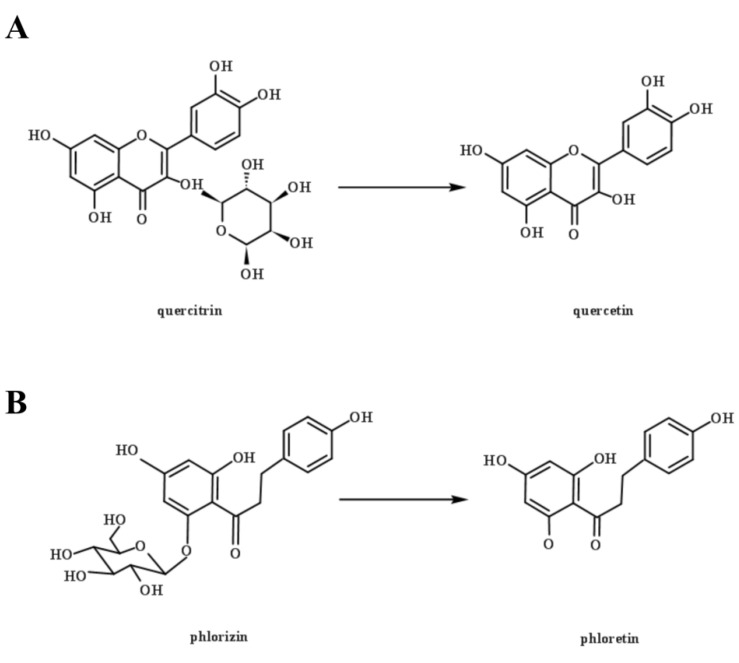
Chemical schematic diagram of quercetin (**A**) and phlorizin (**B**) hydrolyzed by β-glucosidase.

The contents of phenolic profiles differ among different varieties of apples, and between the pulp and the peel [30,31]. A total of four phenolic acids and four flavonoids were identified and quantified in this study (Table 1). *L. rhamnosus* L08 showed better convertibility of the bound polyphenols containing glycosides in apple pomace. Quercitrin and phlorizin were the dominant bound phenolics in apple pomace in this study, with contents of 34.11 and 3.43 μg/mL, respectively. They are flavonoid glycosides formed from their aglycones (quercetin and phloretin) with different types of carbohydrates [10]. LAB fermentation metabolizes phenolic compounds mainly through enzymatic hydrolysis, which converts flavonoid glycosides to the corresponding aglycones (Figure 5), and degrades methyl gallate or tannins [32]. *L. rhamnosus* L08 fermentation significantly reduced the content of quercitrin and phlorizin by 19.78% and 71.72% (*p* < 0.05), whereas it increased the content of quercetin and phloretin by 10.85-fold and 1.94-fold, respectively. This result suggests that the glycosidase produced by *L. rhamnosus* L08 has a much higher affinity for phlorizin than quercitrin. This difference may be due to differences in their molecular structure, particularly their aglycones [10].

In the present work, *L. rhamnosus* L08 fermentation significantly increased the contents of gallic acid, epicatechin acid, caffeic acid, and ferulic acid in apple pomace (*p* < 0.05), indicating that *L. rhamnosus* L08 also has a certain effect on phenolic acid biotransformation. The increase in phenolic acid contents may be partly attributed to the release of bound phenolic acid and partly to the conversion of phenolic acid [10,33]. In addition, the metabolites of LAB and the nutrients in the fermentation substrate could also affect the decarboxylation of free phenolic acids during the fermentation process [28,34].

### 3.4. Changes in Antioxidant Activities during Fermentation

**Figure 6:**
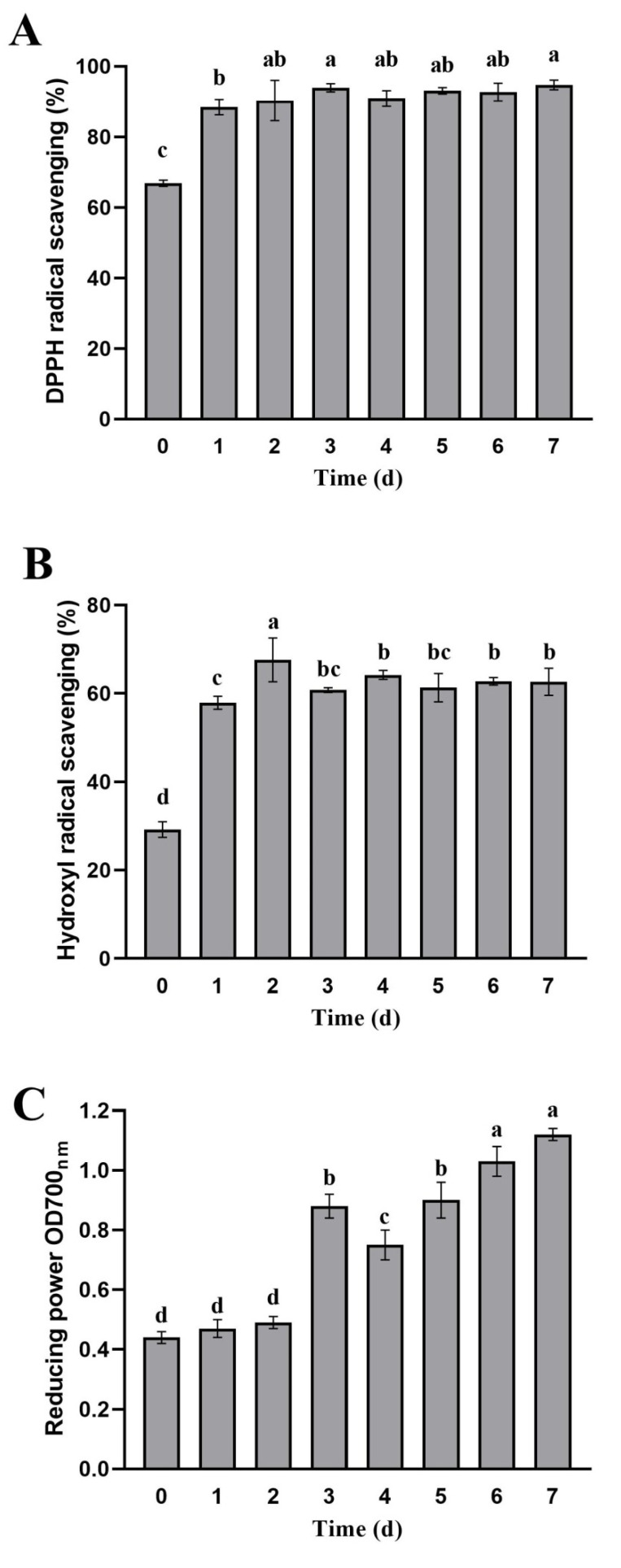
Antioxidant activities of apple pomace during fermentation. (**A**) DPPH radical scavenging activity; (**B**) hydroxyl radical scavenging activity; (**C**) reducing power. Data are means ± SD of three biological replicates. Different letters above bars indicate significant differences (*p* < 0.05).

We evaluated the antioxidant activities of apple pomace fermented by *L. rhamnosus* L08 based on DPPH radical scavenging ability, hydroxyl radical scavenging ability, and reducing power (Figure 6). A significant improvement of the DPPH radical scavenging ability (above 90%) was observed throughout the fermentation process, increasing by 32% at least, in comparison with unfermented apple pomace (*p* < 0.05), but showing no obvious difference during 1 to 7 days of apple pomace fermentation (*p* > 0.05) (Figure 6A). Similar trends were noted in hydroxyl radical scavenging ability results. The hydroxyl radical scavenging ability in apple pomace increased by 1.14-fold on average after *L. rhamnosus* L08 fermentation compared to unfermented apple pomace (*p* < 0.05) (Figure 6B). With the extension of the fermentation time, the reducing power gradually increased. The highest reducing power in apple pomace was observed at 7 days of fermentation, increasing by 1.55-fold as compared with unfermented apple pomace (*p* < 0.05) (Figure 6C).

The positive effects of LAB fermentation on antioxidant activity can be explained by the biotransformation of phenolic profiles and the contribution of each phenolic compound [16]. Although several phenolic compounds possess similar structural features, their antioxidant capacity could differ due to the different number and position of hydroxyl groups, which donate electrons or hydrogen atoms to free radicals [35]. The correlation analysis between phenolic profiles in apple pomace after fermentation and antioxidant activities are shown in Table 2. Gallic acid content was significantly positively correlated with DPPH radical scavenging activity (r = 0.765, *p* < 0.05) and reducing power (r = 0.892, *p* < 0.01), respectively. There was significantly positive correlation between DPPH radical scavenging activity and epicatechin acid (r = 0.863, *p* < 0.01), caffeic acid (r = 0.971, *p* < 0.01), ferulic acid (r = 0.781, *p* < 0.05) in apple pomace after fermentation, and similar correlations were noted in the hydroxyl radical scavenging ability. The reducing power was negatively correlated with the contents of quercitrin (r = −0.956) and phlorizin (r = −0.969), whereas it was positively correlated with the contents of quercetin (r = 0.927) and phloretin (r = 0.972) (*p* < 0.01), respectively. Lu and Foo [36] evaluated the antioxidant properties of chlorogenic acid, epicatechin, quercitrin, and quercetin aglycone isolated from apple pomace, and found that these compounds showed strong antioxidant activities. The presence of more quercetin glycosides in apple peels contributed to its greater antioxidant activity than that found in the flesh [37]. In our study, *L. rhamnosus* L08 fermentation improved the antioxidant activity of apple pomace, which could be the result of the bioconversion of phenolic profiles.

## 4. Conclusions

In this work, *L. rhamnosus* L08 showed better convertibility of the bound polyphenols containing glycosides in apple pomace, compared to other strains. *L. rhamnosus* L08 fermentation significantly increased the total phenols and enhanced the antioxidant activity in apple pomace. Our results demonstrate that LAB fermentation is a promising way to improve the bioactivity of phenolic compounds in apple pomace, although further studies are needed to explore in depth the effect of LAB on phenolic metabolism. Furthermore, more investigations could to be considered for other beneficial constituents (e.g., pectins and cellulose) in apple pomace to improve its utilization value and applications.

## Figures and Tables

**Table 1:** Phenolic profiles in apple pomace during fermentation (μg/mL).

Fermentation Time (d)	Phenolic Acids				Flavonoid Glycosides		Flavonoid Aglycones	
	Gallic Acid	Epicatechin Acid	Caffeic Acid	Ferulic Acid	Quercitrin	Phlorizin	Quercetin	Phloretin
0	0.10 ± 0.08 ^a^	0.40 ± 0.02 ^a^	0.62 ± 0.03 ^a^	0.83 ± 0.05 ^a^	34.11 ± 0.04 ^f^	3.43 ± 0.03 ^g^	0.54 ± 0.04 ^a^	1.05 ± 0.05 ^a^
1	0.15 ± 0.02 ^a^	1.27 ± 0.03 ^b^	0.98 ± 0.04 ^b^	2.76 ± 0.06 ^d^	35.57 ± 0.02 ^g^	3.43 ± 0.04 ^g^	0.54 ± 0.06 ^a^	1.10 ± 0.02 ^a^
2	1.26 ± 0.11 ^b^	1.50 ± 0.04 ^c^	0.98 ± 0.03 ^b^	2.93 ± 0.05 ^e^	35.61 ± 0.05 ^g^	3.04 ± 0.06 ^f^	0.55 ± 0.03 ^a^	1.35 ± 0.06 ^b^
3	2.00 ± 0.22 ^c^	1.93 ± 0.04 ^e^	1.12 ± 0.07 ^c^	2.71 ± 0.02 ^d^	32.28 ± 0.06 ^e^	1.87 ± 0.04 ^e^	3.22 ± 0.04 ^b^	2.38 ± 0.03 ^c^
4	1.37 ± 0.02 ^b^	1.95 ± 0.08 ^e^	1.00 ± 0.05b ^c^	2.73 ± 0.05 ^d^	31.60 ± 0.02 ^d^	1.67 ± 0.02 ^d^	5.44 ± 0.02 ^c^	2.46 ± 0.03 ^c^
5	1.99 ± 0.08 ^c^	1.69 ± 0.04 ^d^	1.08 ± 0.05b ^c^	1.71 ± 0.03 ^b^	30.28 ± 0.02 ^c^	1.38 ± 0.05 ^c^	5.45 ± 0.01 ^c^	2.61 ± 0.02 ^d^
6	2.08 ± 0.18 ^c^	2.29 ± 0.10 ^f^	1.05 ± 0.06b ^c^	2.63 ± 0.07 ^d^	28.99 ± 0.07 ^b^	1.21 ± 0.01 ^b^	6.00 ± 0.01 ^d^	2.69 ± 0.04 ^d^
7	2.05 ± 0.10 ^c^	2.69 ± 0.06 ^g^	1.01 ± 0.03b ^c^	2.45 ± 0.05 ^c^	27.36 ± 0.04 ^a^	0.97 ± 0.03 ^a^	6.40 ± 0.03 ^e^	3.09 ± 0.04 ^e^

Results are presented as the means ± SD of three independent experiments. The superscript a–g followed by means in each time point indicate significant differences at *p* < 0.05. Statistical significance was determined using a one-way ANOVA.

**Table 2:** Correlation between phenolic profiles in apple pomace after fermentation and antioxidant activities.

	DPPH-SA	OH-SA	RPA
Gallic acid	0.765 *	0.649	0.892 **
Epicatechin acid	0.863 **	0.766 *	0.876 **
Caffeic acid	0.971 **	0.896 **	0.607
Ferulic acid	0.781 *	0.865 **	0.197
Quercetin	0.590	0.444	0.927 **
Phloretin	0.681	0.517	0.972 **
Quercitrin	−0.455	−0.267	−0.956 **
Phlorizin	−0.669	−0.514	−0.969 **

DPPH-SA: DPPH radical scavenging ability, OH-SA: hydroxyl radical scavenging ability, RPA: reducing power. The correlation is denoted by r. * Significant correlation at *p* < 0.05, ** Significant correlation at *p* < 0.01.

## Data Availability

Not applicable.
